# Gender Differences in Satisfaction With a Text Messaging Program (Text4Hope) and Anticipated Receptivity to Technology-Based Health Support During the COVID-19 Pandemic: Cross-sectional Survey Study

**DOI:** 10.2196/24184

**Published:** 2021-04-15

**Authors:** Reham Shalaby, Wesley Vuong, Marianne Hrabok, April Gusnowski, Kelly Mrklas, Daniel Li, Mark Snaterse, Shireen Surood, Bo Cao, Xin-Min Li, Russell Greiner, Andrew James Greenshaw, Vincent Israel Opoku Agyapong

**Affiliations:** 1 Department of Psychiatry Faculty of Medicine and Dentistry University of Alberta Edmonton, AB Canada; 2 Addiction and Mental Health Alberta Health Services Edmonton, AB Canada; 3 Cumming School of Medicine University of Calgary Calgary, AB Canada; 4 Strategic Clinical Networks Provincial Clinical Excellence Alberta Health Services Calgary, AB Canada; 5 Department of Community Health Sciences Cumming School of Medicine University of Calgary Edmonton, AB Canada

**Keywords:** COVID-19, Text4Hope, satisfaction, mobile phone, text, anxiety, depression, stress, pandemic, e-mental health, gender

## Abstract

**Background:**

In March 2020, Text4Hope—a community health service—was provided to Alberta residents. This free service aims to promote psychological resilience and alleviate pandemic-associated stress, anxiety, and depression symptoms during the COVID-19 pandemic.

**Objective:**

This study aimed to evaluate the feedback, satisfaction, experience, and perceptions of Text4Hope subscribers and to examine any differences based on gender after subscribers received 6 weeks of daily supportive text messages. Additionally, this study examined subscribers’ anticipated receptivity to technology-based medical services that could be offered during major crises, emergencies, or pandemics.

**Methods:**

Individuals self-subscribed to Text4Hope to receive daily supportive text messages for 3 months. Subscribers were invited to complete a web-based survey at 6 weeks postintervention to provide service satisfaction–related information. Overall satisfaction was assessed on a scale of 0-10, and satisfaction scores were analyzed using a related-measures *t* test. Likert scale satisfaction responses were used to assess various aspects of the Text4Hope program. Gender differences were analyzed using one-way analysis of variance (ANOVA) and Chi-square analyses.

**Results:**

A total of 2032 subscribers completed the baseline and 6-week surveys; 1788 (88%) were female, 219 (10.8%) were male, and 25 (1.2%) were other gender. The mean age of study participants was 44.58 years (SD 13.45 years). The mean overall satisfaction score was 8.55 (SD 1.78), suggesting high overall satisfaction with Text4Hope. The ANOVA analysis, which was conducted using the Welch test (n=1716), demonstrated that females had significantly higher mean satisfaction scores than males (8.65 vs 8.11, respectively; mean difference=0.546; 95% CI 0.19 to 0.91; *P*<.001) and nonsignificantly lower satisfaction scores than other gender respondents (mean difference=−0.938; 95% CI −0.37 to 2.25; *P*=.15). More than 70% of subscribers agreed that Text4Hope helped them cope with stress (1334/1731, 77.1%) and anxiety (1309/1728, 75.8%), feel connected to a support system (1400/1729, 81%), manage COVID-19–related issues (1279/1728, 74%), and improve mental well-being (1308/1731, 75.6%). Similarly, subscribers agreed that messages were positive, affirmative, and succinct. Messages were always or often read by 97.9% (1681/1716) of respondents, and more than 20% (401/1716, 23.4%) always or often returned to messages. The majority of subscribers (1471/1666, 88.3%) read the messages and either reflected upon them or took a positive action. Subscribers welcomed almost all technology-based services as part of their health care during crisis or emergency situations. Text4Hope was perceived to be effective by many female subscribers, who reported higher satisfaction and improved coping after receiving text messages for 6 weeks.

**Conclusions:**

Respondents affirmed the high quality of the text messages with their positive feedback. Technology-based services can provide remotely accessible and population-level interventions that align with the recommended physical distancing practices for pandemics. Text4Hope subscriber feedback revealed high satisfaction and acceptance at 6 weeks postintervention.

**International Registered Report Identifier (IRRID):**

RR2-10.2196/19292

## Introduction

### Background

On March 11, 2020, the World Health Organization declared COVID-19 a global pandemic [1]. By March 23, 2020, there were 332,930 COVID-19 cases worldwide and 14,509 deaths attributed to the pandemic [2]. On this date, Alberta Health Services (the provincial health authority in Alberta, Canada) launched Text4Hope—a free, mobile, community mental health service that aims to support mental well-being and resilience, improve coping mechanisms, and safeguard against pandemic-associated thoughts in Alberta residents [3]. The service was advertised on the Alberta Health Services and Text4Hope funders’ websites and was launched on March 23, 2020. Thousands of people have signed up for the service, and enrollment continues to increase to date. Text4Hope is a text-based mental health support program that involves daily, evidence-based, cognitive behavioral therapy–derived text messages. These messages were carefully designed to accompany a rapidly evolving health crisis and to be scalable, remotely deliverable, and accessible. They were also designed to be cost-effective for funding organizations and free to subscribers [4]. The Text4Hope program was developed based on lessons from the Text4Mood and Text4Support programs [5,6]. Similar to the Text4Mood program, individual Text4Hope self-subscribers receive daily text messages. However, while the Text4Mood messages were crafted to mainly address anxiety, depression, and general well-being among residents of Northern Alberta, the Text4Hope messages were crafted to predominantly address COVID-19–related stress, anxiety, and depression among all Albertans. In contrast to both Text4Mood and Text4Hope, Text4Support was specifically designed to provide support for the eight most commonly observed addiction and mental health concerns in the Edmonton Zone [6]. In this program, a mental health therapist or psychiatrist sorts clients into 1 of the 8 categories, and patients are enrolled by a coordinator inputting the patients’ mobile phone numbers into a web-based program. Text4Hope fills a service gap in Alberta, as social distancing measures may have resulted in high-risk individuals (from a health perspective) not being able to access addiction and mental health services during the early stage of the pandemic. Text4Hope also offers mental health support to those who might not feel comfortable with in-person contact.

During similar crises, the effective and efficient mobilization of community resources was strongly encouraged to support and properly meet mental health needs and avoid future adverse mental health consequences [7]. During pandemics, negative thoughts accompanied by growing uncertainties can pose a threat to personal health and mental well-being. The transmissibility of SARS-CoV-2 has been shown to exceed that of similar viruses (eg, MERS-CoV [Middle East respiratory syndrome coronavirus], H1N1, and SARS-CoV [severe acute respiratory syndrome coronavirus]) [8]. As such, strict policies and regulations were enforced to contain viral spread, including physical distancing, self-isolation, quarantine, travel restrictions, the closure of public schools, and disinfection protocols. However, these measures have likely contributed to mental strain and psychological distress during the COVID-19 pandemic [9,10]. Other iterations of texting programs were developed to support patients with major depressive disorders [11] and alcohol use disorder [12,13]. Individuals in these programs reported an improvement in depression scores and felt better supported in their attempts to quit drinking alcohol after receiving text messages [12,14]. Supportive text messaging services can be tailored to meet the needs of diverse populations. For example, Text4baby and Quit4baby are two services that are provided to pregnant women in the United States [15,16], while Text4Mood and Text4Support are mental health services that are provided to people in Canada [5,6]. Ultimately, such services provide people with hopefulness and support and aim to close the psychological treatment gap in health care systems [5].

To make the best use of resources and enhance the use of texting technology as part of routine practice in health care, it is essential to assess user satisfaction and better understand subscribers’ experiences. The assessment of user satisfaction is a quality method that affects client retention and clinical outcomes [17]. In the customer service industry, relative satisfaction and customer expectations are considered critical components for guaranteeing customer loyalty [18]. In health care systems, self-reported continuity of care strongly correlates with client satisfaction. A recent study has demonstrated that a 7.2% reduction in the frequency of reporting “at least good overall satisfaction” was associated with a 1% increase in hospital bed occupancy [17]. Generally, asynchronous web-based and text-based services have been accepted by an increasing number of individuals who perceive such services as supportive and promising [19]. Most of these programs have usually stated that more than 85% of text message recipients report high satisfaction, high convenience, easy use, and better control over life activities, while above 90% report increased life productivity after receiving text messages [20,21]. Additionally, telephone services are frequently associated with having lower attrition rates than face-to-face services, which is likely due to the accessibility provided by technology that removes geographical barriers. This is especially helpful to those who are tentative about seeking medical attention or require medications [22]. Agyapong and colleagues [5], who evaluated Text4Mood, found that 80% of participants agreed that asynchronous supportive text messages should be provided during follow-up care, and approximately 50% of participants agreed to the use of videoconferencing consultations. A number of variables may affect users’ satisfaction with texting services, such as sociodemographic characteristics, health status, and disease severity. Similarly, one's gender identity may be an important determinant of service acceptability and satisfaction. However, it should be noted that inconsistent findings have been reported for gender identity effects. Although females are highly accepting of surveys and have a high desire to respond to surveys that are delivered to them via a texting service [23], in a feasibility study, the high fidelity of a texting service program was also reported when the program was provided to a group of disadvantaged men at risk of substance or alcohol abuse [24]. In yet another study, authors found no difference between male and female university students in terms of their satisfaction with texting services for alcohol use intervention [14]. Additionally, the initial reports of our program revealed that a majority of our subscribers reported their gender as female (86.9%). This overrepresentation of females in text messaging services has necessitated investigations into user satisfaction and anticipated agreement to receiving technology-based medical services based on gender. Such investigations will allow targeted gender-based interventions to be developed in accordance with user preferences.

This study occurred in Alberta, the Canadian Province where the Text4Hope program was launched. As of July 1, 2020, Alberta had a population of 4,421,876 people, with 68% of the population aged between 15 and 64 years. Alberta has consistently consisted of more males than females (101 males per 100 females), mainly due to the large proportion of working-age males migrating to the province [25]. In 2006, the racial and ethnic composition of Alberta was 80.3% White Canadians, 13.9% visible minority groups, and 5.8% Indigenous groups (3% First Nations, 2.6% Metis, and 0.1% other Indigenous groups). Visible minority groups included the following: Chinese (3.7%), South Asian (3.2%), Filipino (1.6%), Black (1.4%), Southeast Asian (0.9%), Latin American (0.8%), Arab (0.8%), Korean (0.4%), West Asian (0.3%), and Japanese 0.3% [26]. In 2016, more than half (54%) of Canadians aged 25-64 years had either college or university qualifications (an increase from the 48.3% in 2006) [27]. Alberta's gross domestic product at basic prices was CAN $334.5 billion (US $265.2 billion) in 2019 (largely unchanged from Alberta’s gross domestic product in 2018) [28].

### Objective

The aim of this study was to evaluate subscribers’ overall satisfaction with Text4Hope; obtain feedback about subscribers’ experiences and the impact of the texting intervention; explore the perceptions of subscribers about their anticipated receptivity toward diverse, technology-based medical services that are offered as a part of their health care during major crises, emergencies, or pandemics (such as the COVID-19 pandemic); and examine any differences that are based on gender after subscribers received 6 weeks of daily supportive text messages.

### Hypotheses

Based on previous Text4Mood research [5], our hypotheses were as follows: (1) the mean overall satisfaction level with Text4Hope would be at least 7.5 (75%) and (2) at least 75% of subscribers would express anticipated agreement with receiving diverse, technology-based medical services during crises or emergencies. Additionally, we believed that there would be a difference in the satisfaction measure based on the self-declared gender identity of the respondents.

## Methods

### Study Design

This cross-sectional study assessed subscribers’ satisfaction and experiences with Text4Hope and their perceptions of technology-based support after they received 6 weeks of daily text messages.

**Data Collection**

The data collection methods were fully described in the study protocol [29]. In summary, subscribers joined the Text4Hope program [3] and received daily supportive text messages for 3 months by texting the word “COVID19HOPE” to a short code number. The messages were in line with a cognitive behavioral framework that addressed the aspects of potential stresses, anxiety, and depression, and the content was written by mental health professionals. Text message delivery was unidirectional and not specifically tailored to the end users. The following are examples of the messages that were sent:

When bad things happen that we can’t control, we often focus on the things we can’t change. Focus on what you can control; what you can do to help yourself (or someone else) today.

Example 1

What lies behind you and what lies before you are tiny matters compared to what lies within you. Have faith in yourself and success can be yours.

Example 2

Set goals for today, even if they are small. Goals should be “SMART”: Specific, Measurable, Achievable, Realistic, and Timely.

Example 3

The messages were uploaded to a web-based platform, which delivered automated messages at 9 AM. The first message welcomed subscribers to the service and invited them to voluntarily complete a web-based baseline survey, which was used to capture demographic and clinical information that primarily pertained to anxiety, stress, depression, and self-isolation. At 6 weeks postintervention, subscribers were invited (via a text message link) to complete a follow-up web-based survey.

The 6-week survey included standardized scales that were used for the Text4Hope baseline assessments [30,31] as well as an adopted version of the Text4Mood user satisfaction survey [5]. Each survey took 5-10 minutes to complete. No incentives were offered to respondents for completing the baseline or 6-week surveys. Consent was implied if participants clicked on the survey links and submitted their responses.

Participation in the program was voluntary, and the receipt of supportive text messages was not contingent on survey completion. Subscribers could opt out of Text4Hope at any time by texting the word “STOP” to a short code number.

**Figure 1:**
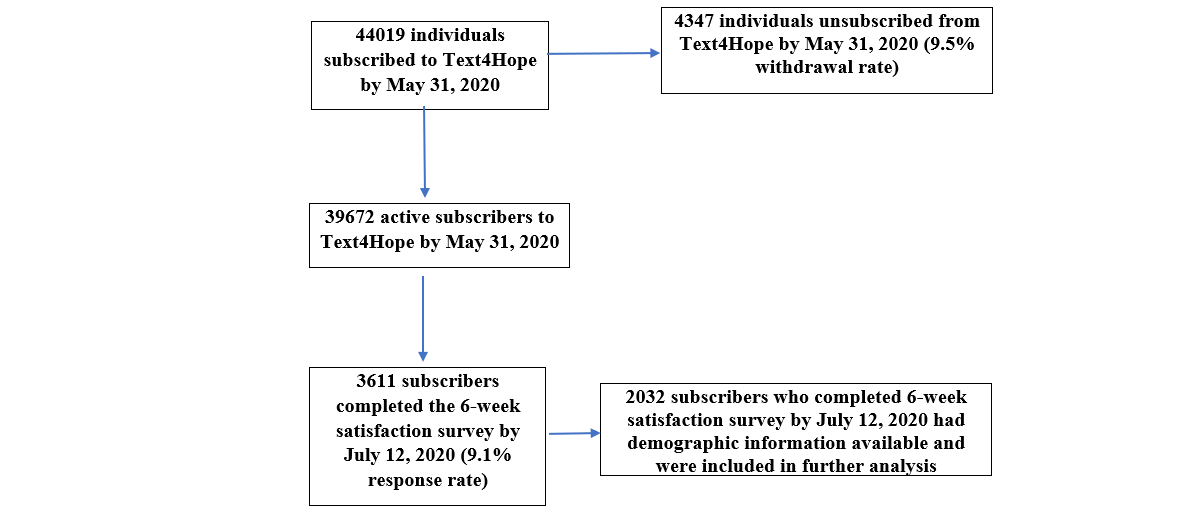
Subscription flowchart.

Six-week satisfaction data were collected between May 31 and July 12, 2020. Figure 1 depicts a subscriber flowchart, which indicates the number of subscribers who completed the web-based surveys at each time point.

The study protocol [29] was approved by the Research and Ethics Board of the University of Alberta (approval number: Pro00086163).

### Outcome Measures

The primary outcome measure was subscribers’ overall satisfaction with the Text4Hope daily supportive text messages. Overall satisfaction at 6 weeks postintervention was based on an 11-point Likert scale (0=very dissatisfied; 5=neither satisfied nor dissatisfied; 10=very satisfied). This overall satisfaction score allowed us to determine whether people liked texting-based services. If people are satisfied with the population-based services they receive, then the services are potentially feasible and can aid in future service planning during pandemics. The satisfaction scale has been used to compare service satisfaction across all addiction and mental health services in the Edmonton Zone. The reliability and validity of this scale has not been tested, although it has been in use for several years.

Secondary outcomes included the perceived impacts of and subscribers’ feedback for the daily supportive text messages at 6 weeks postintervention as well as subscribers’ anticipated receptivity to diverse, technology-based medical services (eg, telephone, videoconferencing, and email for health care) during the COVID-19 pandemic. Gender differences in both primary and secondary measures constituted the exploratory outcome measures.

### Sample Size Considerations

In total, 44,019 individuals were subscribed to Text4Hope in May 31, 2020. We estimated that a sample size of 1775 was needed to estimate the overall mean satisfaction rate (based on an 11-point scale from 0 to 10) for the entire population with a 3% margin of error and 99% confidence.

### Analysis

Data were analyzed using SPSS Statistics for Windows, version 26 (IBM Corporation) [32]. Demographic characteristics were summarized as raw numbers and percentages. We measured subscribers’ overall satisfaction on an 11-point Likert scale (0=very dissatisfied; 5=neither satisfied nor dissatisfied; 10=very satisfied) and analyzed responses by using the related sample *t* test. We explored gender differences in satisfaction, which was measured on the same scale, by using one-way analysis of variance (ANOVA) tests. A Bonferroni-corrected, two-tailed criterion (α<.002) was used to determine statistical differences. Likert scale satisfaction responses to various aspects of Text4Hope and anticipated receptivity to technology-based interventions (web-based counseling, telephone counseling, text and email messaging, telephone consultations for physical and mental health, and video consultations for physical and mental health) were summarized as frequency counts of response categories and percentages. We compared gender differences in satisfaction and preferences for technology-based interventions by using the Fisher exact test with two-tailed, Bonferroni-corrected criteria for 23 variables (α<.002) to determine statistical differences. There was no imputation for missing data, and the results were based on completed survey responses.

Between May 31 and July 12, 2020, 39,672 active Text4Hope subscribers were invited to complete the 6-week survey. Of these subscribers, 3611 completed the survey, yielding a response rate of 9.1%. Of the 2032 subscribers who had available demographic information from their baseline survey and were included in further analysis, 1788 (88%) were female, 219 (10.8%) were male, and 25 (1.2%) were other gender. Table 1 provides a descriptive analysis of the demographics of respondents.

**Table 1:** Demographic and clinical characteristics of respondents at 6 weeks postintervention.

Variables		Male (n=219), n (%)	Female (n=1788), n (%)	Other gender (n=25), n (%)	Overall (N=2032), n (%)
**Age (years)**					
	≤25	15 (7)	151 (8.6)	7 (28)	173 (8.6)
	26-40	53 (24.8)	490 (27.7)	12 (48)	555 (27.7)
	41-60	105 (49.1)	891 (50.5)	4 (16)	1000 (49.9)
	>60	41 (19.2)	234 (13.3)	2 (8)	277 (13.8)
**Ethnicity**					
	White	177 (81.2)	1492 (83.9)	19 (76)	1688 (83.5)
	Indigenous	6 (2.8)	54 (3)	0 (0)	60 (3)
	Asian	15 (6.9)	90 (5.1)	1 (4)	106 (5.2)
	Other	20 (9.2)	142 (8)	5 (20)	167 (8.3)
**Education**					
	Less than a high school diploma	7 (4)	35 (2.3)	2 (8.7)	44 (2.6)
	High school diploma	14 (8)	102 (6.8)	1 (4.3)	117 (6.9)
	Postsecondary education	155 (88.1)	1349 (90.2)	19 (82.6)	1523 (89.9)
	Other education	0 (0)	9 (0.6)	1 (4.3)	10 (0.6)
**Employment status**					
	Employed	120 (69)	1059 (71.5)	12 (52.2)	1191 (70.9)
	Unemployed	26 (14.9)	177 (11.9)	3 (13)	206 (12.3)
	Retired	23 (13.2)	151 (10.2)	2 (8.7)	176 (10.5)
	Student	4 (2.3)	71 (4.8)	5 (21.7)	80 (4.8)
	Other	1 (0.6)	24 (1.6)	1 (4.3)	26 (1.5)
**Relationship status**					
	Married/cohabiting/partnered	112 (63.3)	987 (66)	11 (47.8)	1110 (65.5)
	Separated/divorced	14 (7.9)	154 (10.3)	1 (4.3)	169 (10)
	Widowed	3 (1.7)	37 (2.5)	1 (4.3)	41 (2.4)
	Single	46 (26)	303 (20.3)	9 (39.1)	358 (21.1)
	Other	2 (1.1)	14 (0.9)	1 (4.3)	17 (1)
**Housing status**					
	Own home	122 (69.7)	1037 (70.1)	12 (52.2)	1171 (69.8)
	Living with family	14 (8)	132 (8.9)	4 (17.4)	150 (8.9)
	Renting	38 (21.7)	300 (20.3)	5 (21.7)	343 (20.5)
	Other	1 (0.6)	10 (0.7)	2 (8.7)	13 (0.8)

## Results

### Demographic and Clinical Characteristics

Table 1 displays subscribers’ demographic characteristics based on different genders. The data indicated that most respondents were aged between 26 and 60 years (1555/2032, 77.6%); were White (1688/2032, 83.5%); were married, cohabiting, or partnered (1110/2032, 65.5%); reported the completion of postsecondary education (1523/2032, 89.9%); were employed (1191/2032, 70.9%); and owned their own home (1171/2032, 69.8%).

### Primary Outcome Measure

Respondents were asked to rate their overall satisfaction with the daily supportive text messaging (Text4Hope) service on a scale of 0-10, in which 0 represented “very dissatisfied,” 5 represented “neither satisfied nor dissatisfied,” and 10 represented “very satisfied.” Respondents’ (n=2940) mean overall satisfaction score was 8.55 (SD 1.78), suggesting that overall, respondents’ satisfaction with the Text4Hope program was high. The ANOVA analysis, which was conducted using the Welch test (n=1716), demonstrated that females had significantly higher mean satisfaction scores than males (8.65 vs 8.11, respectively; mean difference=0.546; 95% CI 0.19 to 0.91; *P*<.001) and nonsignificantly lower satisfaction scores than other gender respondents (mean difference=−0.938; 95% CI −0.37 to 2.25; *P*=0.15).

### Secondary Outcome Measures

In Table 2, we show subscribers’ level of agreement regarding Text4Hope benefits. This table displays the perceived impact of Text4Hope messages after subscribers received daily text messages for 6 weeks.

**Table 2:** Gender differences in the perceived impact of daily messages at 6 weeks postintervention.

Perceived impact of daily messages from Text4Hope		Male, n (%)	Female, n (%)	Other gender, n (%)	*P* value^a^	Total, n (%)
**Helped subscribers cope with stress related to the COVID-19 pandemic**						
	Agree	144 (75.8)	1177 (77.4)	13 (61.9)	.05	1334 (77.1)
	Neutral	33 (17.4)	284 (18.7)	5 (23.8)	N/A^b^	322 (18.6)
	Disagree	13 (6.8)	59 (3.9)	3 (14.3)	N/A	75 (4.3)
**Helped subscribers cope with anxiety related to the COVID-19 pandemic**						
	Agree	133 (70.4)	1162 (76.5)	14 (66.7)	.05	1309 (75.8)
	Neutral	44 (23.3)	297 (19.6)	4 (19)	N/A	345 (20)
	Disagree	12 (6.3)	59 (3.9)	3 (14.3)	N/A	74 (4.3)
**Helped subscribers cope with depression related to the COVID-19 pandemic**						
	Agree	103 (54.5)	856 (56.4)	9 (42.9)	.04	968 (56.1)
	Neutral	63 (33.3)	561 (37)	9 (42.9)	N/A	633 (36.7)
	Disagree	23 (12.2)	100 (6.6)	3 (14.3)	N/A	126 (7.3)
**Helped subscribers cope with loneliness related to the COVID-19 pandemic**						
	Agree	71 (37.4)	757 (49.9)	9 (42.9)	.01	837 (48.5)
	Neutral	85 (44.7)	592 (39.1)	9 (42.9)	N/A	686 (39.7)
	Disagree	34 (17.9)	167 (11)	3 (14.3)	N/A	204 (11.8)
**Made subscribers feel connected to a support system during the COVID-19 pandemic**						
	Agree	144 (75.8)	1242 (81.8)	14 (66.7)	.05	1400 (81)
	Neutral	33 (17.4)	211 (13.9)	4 (19)	N/A	248 (14.3)
	Disagree	13 (6.8)	65 (4.3)	3 (14.3)	N/A	81 (4.7)
**Made subscribers feel hopeful about managing issues related to the COVID-19 pandemic**						
	Agree	134 (70.5)	1133 (74.7)	12 (57.1)	.09	1279 (74)
	Neutral	46 (24.2)	324 (21.4)	6 (28.6)	N/A	376 (21.8)
	Disagree	10 (5.3)	60 (4)	3 (14.3)	N/A	73 (4.2)
**Improved subscribers’ overall mental well-being**						
	Agree	136 (71.6)	1159 (76.2)	13 (61.9)	.06	1308 (75.6)
	Neutral	38 (20)	289 (19)	5 (23.8)	N/A	332 (19.2)
	Disagree	16 (8.4)	72 (4.7)	3 (14.3)	N/A	91 (5.3)
**Enhanced subscribers’ quality of life**						
	Agree	104 (55)	941 (62.5)	12 (60)	.11	1057 (61.7)
	Neutral	68 (36)	474 (31.5)	5 (25)	N/A	547 (31.9)
	Disagree	17 (9)	90 (6)	3 (15)	N/A	110 (6.4)

^a^Bonferroni-corrected, two-tailed criteria for significance (α<.002).

^b^N/A: not applicable.

The results in Table 2 indicate that about three-quarters of respondents agreed that the daily text messages helped them cope with stress (1334/1731, 77.1%) and anxiety (1309/1728, 75.8%) as well as manage COVID-19–related issues (1279/1728, 74%), while about half of the respondents agreed that the messages helped them cope with depression (968/1727, 56.1%) and loneliness (837/1727, 48.5%). About 80% of respondents agreed that they felt connected to a support system due to receiving the daily messages (1400/1729, 81%), a little over 70% of respondents agreed that the daily messages helped to improve their mental well-being (1308/1731, 75.6%), and about 60% of respondents agreed that the daily messages helped to enhance their quality of life (1057/1714, 61.7%). Overall, compared to males and respondents of other gender identities, a higher proportion of females agreed with all Text4Hope benefits; however, there were no statistically significant gender differences in the levels of agreement expressed for all areas assessed.

Table 3 describes subscribers’ opinions about Text4Hope messages after they received 6 weeks of daily text messages. The data indicated that about three-quarters of respondents always found the Text4Hope text messages to be positive (1336/1732, 77.1%), affirmative (1231/1727, 71.3%), and succinct (1254/1722, 72.8%). More than 80% of respondents (1505/1753, 87.4%) indicated that the messages were always or often relevant. Again, compared to males and respondents of other gender identities, a higher proportion of females reported that they found the messages to be always positive, affirmative, succinct, and relevant (*P<*.001 for each posthoc comparison using z-scores).

Most respondents (1531/1716, 89.2%) indicated that they always read the text messages, and about 20% of respondents indicated that they always or often returned to read the text messages (401/1716, 23.4%). Neither factor indicated gender differences upon analysis. Table 3 data shows that slightly more than 70% respondents (1270/1666, 76.2%) indicated that they read and reflected on the text messages, while about 10% of respondents indicated that they took positive or beneficial actions after reading the text messages (201/1666, 12.1%). Although not statistically significant (*P*=.003), compared to males and respondents of other gender identities, a higher proportion of females indicated that they read the text messages, reflected on the messages, and took positive or beneficial actions after reading the messages. No subscribers indicated that they read the messages and took a negative action.

**Table 3:** Gender differences in the feedback about Text4Hope messages at 6 weeks postintervention.

Feedback		Male, n (%)	Female, n (%)	Other gender, n (%)	*P* value^a^	Total, n (%)
**Text4Hope text messages were positive**						
	Always	131 (68.9)	1193 (78.4)	12 (57.1)	<.001	1336 (77.1)
	Often	55 (28.9)	291 (19.1)	6 (28.6)	N/A^b^	352 (20.3)
	Sometimes	3 (1.6)	35 (2.3)	2 (9.5)	N/A	40 (2.3)
	Rarely	1 (0.5)	2 (0.1)	1 (4.8)	N/A	4 (0.2)
	Never	0 (0)	0 (0)	0 (0)	N/A	0 (0)
**Text4Hope text messages were affirmative**						
	Always	118 (62.8)	1104 (72.7)	9 (42.9)	<.001	1231 (71.3)
	Often	57 (30.3)	347 (22.9)	10 (47.6)	N/A	414 (24)
	Sometimes	11 (5.9)	58 (3.8)	1 (4.8)	N/A	70 (4.1)
	Rarely	1 (0.5)	8 (0.5)	0 (0)	N/A	9 (0.5)
	Never	1 (0.5)	1 (0.1)	1 (4.8)	N/A	3 (0.2)
**Text4Hope text messages were succinct**						
	Always	128 (67.7)	1114 (73.7)	12 (57.1)	.09	1254 (72.8)
	Often	49 (25.9)	300 (19.8)	5 (23.8)	N/A	354 (20.6)
	Sometimes	12 (6.3)	92 (6.1)	4 (19)	N/A	108 (6.3)
	Rarely	0 (0)	6 (0.4)	0 (0)	N/A	6 (0.3)
	Never	0 (0)	0 (0)	0 (0)	N/A	0 (0)
**Text4Hope text messages were relevant**						
	Always	95 (50.5)	945 (62.4)	12 (57.1)	<.001	1052 (61.1)
	Often	64 (34)	386 (25.5)	3 (14.3)	N/A	453 (26.3)
	Sometimes	20 (10.6)	163 (10.8)	3 (14.3)	N/A	186 (10.8)
	Rarely	7 (3.7)	19 (1.3)	2 (9.5)	N/A	28 (1.6)
	Never	2 (1.1)	1 (0.1)	1 (4.8)	N/A	4 (0.2)
**Subscribers’ frequency of reading messages**						
	Always	161 (84.7)	1351 (89.8)	19 (90.5)	.61	1531 (89.2)
	Often	23 (12.1)	125 (8.3)	2 (9.5)	N/A	150 (8.7)
	Sometimes	5 (2.6)	25 (1.7)	0 (0)	N/A	30 (1.7)
	Rarely	1 (0.5)	2 (0.1)	0 (0)	N/A	3 (0.2)
	Never	0 (0)	2 (0.1)	0 (0)	N/A	2 (0.1)
**Subscribers’ frequency of returning to messages**						
	Always	7 (3.7)	73 (4.9)	0 (0)	.47	80 (4.7)
	Often	33 (17.4)	287 (19.1)	1 (4.8)	N/A	321 (18.7)
	Sometimes	76 (40)	635 (42.2)	13 (61.9)	N/A	724 (42.2)
	Rarely	46 (24.2)	327 (21.7)	4 (19)	N/A	377 (22)
	Never	28 (14.7)	183 (12.2)	3 (14.3)	N/A	214 (12.5)
**Actions taken by subscribers after reading text messages**						
	Read text and took a positive or beneficial action	14 (7.7)	186 (12.7)	1 (5)	.003	201 (12.1)
	Read text and reflected on the messages	138 (75.4)	1119 (76.5)	13 (65)	N/A	1270 (76.2)
	Read the text and took no action	25 (13.7)	138 (9.4)	6 (30)	N/A	169 (10.1)
	Read text and took a negative or harmful action	0 (0)	0 (0)	0 (0)	N/A	0 (0)
	Did not read the text	2 (1.1)	2 (0.1)	0 (0)	N/A	4 (0.2)
	Other	4 (2.2)	18 (1.2)	0 (0)	N/A	22 (1.3)

^a^Bonferroni-corrected, two-tailed criteria for significance (α<.002).

^b^N/A: not applicable.

We explored subscribers’ anticipated receptivity to welcoming diverse, technology-based services as part of their health care during crisis or emergency situations, such as the COVID-19 pandemic. The results displayed in Table 4 suggest that at least 80% of respondents agreed with receiving web-based counseling (1390/1674, 83%), telephone counseling (1346/1672, 80.5%), and text messages (1465/1669, 87.8%) as part of their health care during any crisis or emergency situation, such as the COVID-19 pandemic. There were no gender differences in respondents’ preferences for welcoming web-based counseling, telephone counseling, and text messaging as part of their health care during any crisis or emergency situation. Similarly, about 70% of respondents agreed with receiving consultations via video and telephone for both physical (video: 1190/1674, 71.1%; telephone: 1193/1665, 71.7%) and mental (video: 1244/1674, 74.3%; telephone: 1245/1669, 74.6%) health care during any crisis or emergency situation, such as the COVID-19 pandemic. There were no gender-based differences in expressed preferences. Finally, about 60% of respondents agreed with receiving email messages as part of their health care during a crisis or emergency situation, such as the COVID-19 pandemic (1084/1669, 64.9%). Compared to female and male respondents, a higher proportion of other gender respondents agreed with receiving email messages as part of their health care during a crisis or emergency situation.

**Table 4:** Anticipated receptivity of subscribers to receiving diverse, technology-based services as part of their health care during crisis or emergency situations, such as the COVID-19 pandemic.

Subscribers’ anticipated receptivity to services		Male, n (%)	Female, n (%)	Other gender, n (%)	*P* value^a^	Total, n (%)
**Subscribers would welcome web-based counseling for stress, anxiety, and depression**						
	Agree	152 (80.9)	1220 (83.3)	18 (85.7)	.55	1390 (83)
	Neutral	26 (13.8)	198 (13.5)	3 (14.3)	N/A^b^	227 (13.6)
	Disagree	10 (5.3)	47 (3.2)	0 (0)	N/A	57 (3.4)
**Subscribers would welcome telephone counseling for stress, anxiety, and depression**						
	Agree	151 (80.3)	1176 (80.4)	19 (90.5)	.80	1346 (80.5)
	Neutral	29 (15.4)	229 (15.7)	2 (9.5)	N/A	260 (15.6)
	Disagree	8 (4.3)	58 (4)	0 (0)	N/A	66 (3.9)
**Subscribers would welcome text messaging for stress, anxiety, and depression**						
	Agree	159 (84.6)	1288 (88.2)	18 (85.7)	.12	1465 (87.8)
	Neutral	19 (10.1)	132 (9)	1 (4.8)	N/A	152 (9.1)
	Disagree	10 (5.3)	40 (2.7)	2 (9.5)	N/A	52 (3.1)
**Subscribers would welcome email messaging for stress, anxiety, and depression**						
	Agree	106 (56.7)	962 (65.8)	16 (76.2)	.01	1084 (64.9)
	Neutral	45 (24.1)	345 (23.6)	4 (19)	N/A	394 (23.6)
	Disagree	36 (19.3)	154 (10.5)	1 (4.8)	N/A	191 (11.4)
**Subscribers would welcome mental health video consultations**						
	Agree	132 (70.2)	1094 (74.7)	18 (85.7)	.29	1244 (74.3)
	Neutral	42 (22.3)	284 (19.4)	1 (4.8)	N/A	327 (19.5)
	Disagree	14 (7.4)	87 (5.9)	2 (9.5)	N/A	103 (6.2)
**Subscribers would welcome physical health video consultations**						
	Agree	119 (63.3)	1055 (72)	16 (76.2)	.12	1190 (71.1)
	Neutral	50 (26.6)	279 (19)	4 (19)	N/A	333 (19.9)
	Disagree	19 (10.1)	131 (8.9)	1 (4.8)	N/A	151 (9)
**Subscribers would welcome mental health telephone consultations**						
	Agree	126 (67.4)	1102 (75.4)	17 (81)	.19	1245 (74.6)
	Neutral	44 (23.5)	259 (17.7)	3 (14.3)	N/A	306 (18.3)
	Disagree	17 (9.1)	100 (6.8)	1 (4.8)	N/A	118 (7.1)
**Subscribers would welcome physical health telephone consultations**						
	Agree	124 (66.3)	1052 (72.2)	17 (81)	.30	1193 (71.7)
	Neutral	44 (23.5)	258 (17.7)	3 (14.3)	N/A	305 (18.3)
	Disagree	19 (10.2)	147 (10.1)	1 (4.8)	N/A	167 (10)

^a^Bonferroni-corrected, two-tailed criteria for significance (α<.002).

^b^N/A: not applicable.

## Discussion

This study provided results regarding subscribers’ satisfaction with Text4Hope after they received the texting intervention for 6 weeks. Our results revealed considerable satisfaction with Text4Hope. The total number of subscribers who completed the baseline and 6-week surveys was 2032, and a majority of subscribers were female (1788/2032, 88%). The mean age of study participants was 44.58 years. Overall service satisfaction was high, and more than 70% of subscribers agreed that Text4Hope helped them cope with stress (1334/1731, 77.1%) and anxiety (1309/1728, 75.8%), feel connected to a support system (1400/1729, 81%), manage COVID-19–related issues (1279/1728, 74%), and improve mental well-being (1308/1731, 75.6%). Similarly, subscribers agreed that the text messages were positive, affirmative, and succinct. Text messages were always or often read by 97.9% (1681/1716) of respondents, and more than 20% (401/1716, 23.4%) always or often returned to messages. Most subscribers (1471/1666, 88.3%) read the messages and either reflected upon them or took a positive action. Subscribers welcomed almost all technology-based services as part of their health care during crisis or emergency situations. Text4Hope was perceived to be effective by more female subscribers than male or other gender subscribers. The withdrawal rate for Text4Hope was approximately 10% at 6 weeks postintervention. Untailored and unilateral texting services often have high withdrawal rates that range from 0% to 57% [14,33]. Additionally, prior studies have reported that withdrawal rates may be higher for people who receive interventions via SMS text messages compared to those for people who receive the same intervention via email [14]. In a review of 93 mental health apps that target anxiety, depression, or emotional well-being, the median 15-day and 30-day app retention rates were only 3.9% (IQR 10.3%) and 3.3% (IQR 6.2%), respectively [34]. It is possible that our Text4Hope program achieved a higher retention rate compared to those of other mental health apps because it is unidirectional and requires no additional effort or action on the part of the subscriber following enrollment. It is also possible that the message content, which was crafted by mental health professionals; the high anxiety, stress, and depression levels that the population has experienced due to the COVID-19 pandemic; and the reduced availability of face-to-face services contributed to the high Text4Hope retention rate.

Female respondents comprised the majority of the sample in our study (1788/2032, 88%). In other texting-based services, females were also highly represented (>80% of participants) [5]. There were obvious gender differences in subscriber satisfaction rates for Text4Hope. Another study, in which 240 university students received a fully automated, multiple-session alcohol intervention, reported that the majority of students were satisfied with the content and length of the texts; no gender-based differences in responses were reported [14].

Subscribers’ overall satisfaction with our provided service (8.55) was high. This is in line with the 95% satisfaction rate of the Text4Mood program reported by Agyapong et al [5]. Similar findings were reported in a review of text message use among a population with mental health concerns [35]. Bendsten and Bendsten [14] previously reported on participant satisfaction (range 57.9%-84.6%) in relation to the frequency, content, and length of messages. Our study results indicated that females were generally more satisfied with the overall program than males. Generally, the relationship between user satisfaction with health services and self-reported gender seems inconclusive. In a systematic review of 39 studies, the majority of the studies (66.7%) showed that there was no significant relationship between the two factors, and the rest were nearly equally divided in terms of favoring either males or females [36].

Self-reported levels of the ability to cope with psychiatric burdens was mostly lower in Text4Hope respondents than in respondents from the Text4Mood study by Agyapong et al [5]. This was true for respondents with depression (56.1% vs 76.7%) and those who experienced loneliness (48.5% vs 57%). However, our results on participants’ ability to cope with stress symptoms were consistent with those of Agyapong et al [5] (77.1% vs 77.2%). These differences could be attributed to the unprecedented COVID-19 pandemic, associated distress, and the strict pandemic-related restrictions (eg, self-isolation and quarantine). These restrictions may be perceived as limitations of personal freedom and activity and may contribute to feelings of loneliness. Similarly, while the perceived improvement in quality of life scores was positive for more than half of our respondents (1057/1714, 61.7%), it was about 14% lower than that of the Agyapong et al study [5]. This may reflect the potentially high negative and multifocal impacts of the COVID-19 pandemic on people’s perceived quality of life. In addition, females reported high satisfaction with the Text4Hope program’s ability to help them cope with loneliness and depression. This may be in line with the view that depressive symptoms are more frequently experienced by females [37] than males and the fact that people are usually more willing to participate in research that is related to a condition or disease that they have experienced [38]. Text4Hope therefore seems to be a useful support service that helps to ameliorate distressing symptoms in this differentially affected group.

More than 70% of the people in our study reported that the Text4Hope messages were always positive (1336/1732, 77.1%), affirmative (1231/1727, 71.3%), and succinct (1254/1722, 72.8%). About 60% of respondents reported that the messages were always relevant (1052/1753, 61.1%). These results typically came from females, who are usually satisfied with texting services and actively interact with such text messages [19]. Our satisfaction rates were higher than the rates reported by Agyapong et al [5], which ranged from 45.1% to 60%. Similarly, the feeling of being connected to the health care system received higher positive response rates than those in the Agyapong et al study [5] (81% vs 75.2%, respectively). This result may reflect Alberta residents’ true need to connect with a health care system during the absence of the regular, conventional care that was provided before the COVID-19 pandemic, given that all of our subscribers were actively seeking help through the texting program.

The number of Text4Hope respondents who reported that they always or often read the text messages was similar to that of the 2016 Agyapong et al study [5] and higher than that of the 2013 Agyapong et al study [39] (84%). Additionally, more than half our subscribers (1125/1716, 65.6%) reported that they always, often, or sometimes returned to the text messages. This is fairly comparable to the Agyapong et al study [5], wherein 33% of respondents reported that they returned to text messages more than once, with no gender differences observed. This is also consistent with the Bendsten & Bendsten study, which reported no differences based on gender in students’ satisfaction with a texting service for alcohol use disorder [14]. Consistent with the observations in the study by Agyapong et al [5], the majority of our respondents (1471/1666, 88.3%) reported that they reflected on text messages or took positive actions after reading the text messages, and we believe this could be attributed to the reported positive impact of the program on respondents.

With regard to subscribers’ anticipated agreement with the provision of diverse, technology-based medical services, our respondents generally praised the use of these services during the COVID-19 pandemic and other similar crises. Compared to the other proposed technology-based medical services, our results showed that text messaging was the most highly accepted intervention, with an overall agreement rate of 87.8% (1465/1669). This could be explained by the simple nature of such programs, which is important to the end users who usually own cell phones, and by the short and easy-to-read nature of the daily text messages.

Our study reported slightly lower levels of acceptance for video consultation services for both mental and physical health compared to those for web-based counseling services. This may be attributed to the lack of required physical interaction in video consultation services, as the one-way nature of web-based counseling services is usually more accepted and welcomed by users [40]. However, when therapeutic interaction is required, users may prefer face-to-face services, especially in times of global crises, due to privacy concerns related to therapy in the context of web communication [41]. Additionally, the physical presence of a therapist could play a therapeutic role and promote more interaction, subsequently improving resilience and overall psychological outcomes, especially on a long-term basis [39].

This study has several limitations. For instance, there was a low response rate (9.1%) among the 6-week subscribers, which may have been due to the incentive-free and optional nature of the survey. Thus, the reported levels of satisfaction may have been skewed if there was a systematic difference in the measured features between responders and nonresponders. Notwithstanding the low response rate, our sample size exceeded the 1775 respondents needed to estimate satisfaction rates for the entire subscriber population with a 3% margin of error and 99% confidence. Consequently, our study was sufficiently powered to provide satisfaction rate estimates for the entire population of Text4Hope subscribers. Furthermore, Text4Hope has achieved a higher retention rate than those of other mental health apps that target anxiety, depression, or emotional well-being [18,34]. This high retention rate potentially reflects Text4Hope user satisfaction, which may not be captured through surveys for which completion may be considered time-consuming by some subscribers.

It is also possible that we achieved high satisfaction because people who like technology may have been drawn to the Text4Hope program. Additionally, there is potential for social desirability bias, which may have resulted in respondents reporting higher satisfaction and better perceived benefits from receiving text messages. However, this is unlikely due to the anonymous nature of the survey.

There are several other possible limitations. It is possible that our finding that texting was the most accepted mode of delivery for technology-based health services was biased, as those who liked text messaging were likely to sign up for Text4Hope and therefore participate in the survey. It would have been ideal to include a a control group for the comparison of Text4Hope subscribers’ and nonsubscribers’ anticipated receptivity to technology based medical services. Additionally, although there was a statistically significant gender difference in overall satisfaction between males and females (*P*<.001), the magnitude of the difference was very small and unlikely to be practically meaningful, especially given the imbalance of gender identity subsample sizes. Similarly, our study population was skewed toward females, which is not representative of the population in Alberta or Canada. Finally, respondents’ feedback regarding their ability to cope with psychiatric conditions was self-assessed and was not corroborated by clinical assessments.

In conclusion, our results indicate that texting-based programs are acceptable to end users, as high overall satisfaction was reported by subscribers of all gender identities. However, female subscribers reported significantly higher satisfaction scores than male subscribers. Our respondents affirmed the high quality of the text messages by consistently reading and rereading the text messages and providing positive feedback regarding the messages’ supportive nature. Text-based mental health support services can be easily deployed during pandemics to support at-risk populations and alleviate the negative mental health impacts that have been well-documented during uncertain times. Based on Text4Hope subscriber feedback, messages from text-based support interventions that have a 160-character limit, are written by health professionals, and are delivered daily can result in high levels of acceptance and satisfaction upon implementation.

## Abbreviations

ANOVA: analysis of variance
MERS-CoV: Middle East respiratory syndrome coronavirus
SARS-CoV: severe acute respiratory syndrome coronavirus
